# Synthesis and Preclinical Evaluation of 188Re-MAX-HEDP: A Promising Theranostics Strategy for Bone Metastases

**DOI:** 10.5812/ijpr-170436

**Published:** 2026-07-21

**Authors:** Sarah Shoohani, Saeed Kakaei, Elham Sattarzadeh Khameneh, Fariba Johari Daha, Mojtaba Tajik

**Affiliations:** 1 School of Physics, Damghan University, Damghan, Iran; 2 Radiation Application Research School, Nuclear Science and Technology Research Institute, P. O. Box 11365 - 3486, Tehran, Iran

**Keywords:** Radiopharmaceutical, ^188^Re-MAX-HEDP, Bone-Related Diseases, ^188^Re Labeling

## Abstract

**Background:**

Bone-associated disorders, including metastatic bone lesions, osteoporosis, and pathological fractures, require agents that can support both diagnostic imaging and targeted therapy. Theranostic radiopharmaceuticals provide an integrated approach for disease detection and radionuclide therapy.

**Objectives:**

This study aimed to design, synthesize, radiolabel, and evaluate a novel bone-targeting theranostic radiopharmaceutical, ^188^Re-MAX-HEDP, for potential diagnostic and therapeutic applications for bone-related diseases.

**Methods:**

A novel ligand, MAX-HEDP, was synthesized by conjugating hydroxyethylidene diphosphonic acid (HEDP) to the chelating moiety methoxy amido xanthate (MAX). The ligand was radiolabeled with rhenium-188 (^188^Re). Labeling efficiency, chemical yield, in vitro stability under physiological conditions, and biodistribution in normal mice were evaluated.

**Results:**

The synthesis of MAX-HEDP achieved an overall chemical yield of 91%. Radiolabeling with ^188^Re produced a highly stable complex, with a labeling efficiency exceeding 99%. Stability studies showed that more than 96% of the complex remained intact at ambient temperature, whereas degradation at 37 °C was less than 10%. Biodistribution studies demonstrated pronounced skeletal uptake of ^188^Re-MAX-HEDP compared with uptake in other organs, confirming its strong bone-targeting potential.

**Conclusions:**

^188^Re-MAX-HEDP demonstrated a high synthesis yield, excellent radiolabeling efficiency, favorable stability, and selective skeletal accumulation. These findings suggest that it is a promising, cost-effective theranostic candidate for further clinical evaluation in the diagnosis and radionuclide therapy of bone malignancies and other bone-associated disorders.

## 1. Background

The treatment of bone malignancies is challenging because of the complex bone microenvironment and the vascular characteristics of the surrounding tissues. Bone metastases are common in advanced cancers, such as breast, prostate, and lung cancer, and are often associated with severe pain and skeletal-related complications, including fractures and spinal cord compression (1). Targeted drug delivery to bone offers a promising strategy by increasing local drug concentrations while minimizing systemic side effects. Therefore, effective pain palliation and improved disease control are key priorities in the management of patients with metastatic bone disease (2). Notably, combining radiopharmaceuticals and bisphosphonates offers therapeutic benefits by acting as smart drug delivery systems (1). However, further preclinical and clinical evaluations are needed to improve understanding of these combination therapies and their potential translation into clinical practice. Despite scientific advances, studies emphasizing the use of bone scaffolds as local drug delivery systems remain limited (3).

Radioisotopes are chemical elements that undergo radioactive decay. In the context of bone metastases, certain radioisotopes can be used for bone pain palliation and treatment. Radioisotopes have been shown to relieve pain, reduce patients' need for conventional painkillers, improve quality of life, and increase survival (4). The treatment of bone metastases presents significant challenges. Local radiation therapy is an effective method for managing bone pain; however, the development of multiple metastatic sites is common (5). Wide-field, or hemibody, radiotherapy can be effective, but it is often accompanied by substantial bone marrow and gastrointestinal toxicity (6).

In this context, internal radiotherapy using bone-specific local radiopharmaceuticals has emerged as a promising alternative. This approach has been associated with fewer side effects than other treatments (7). Importantly, pain relief with this therapy may result not only from tumor cell killing but also from the death of radiation-sensitive lymphocytes, which secrete various pain-related cytokines at the tumor site (8). This work investigates the therapeutic potential of ^188^Re-based bone-targeted local radiopharmaceuticals for the management of bone metastases and other bone malignancies, drawing on experimental and preclinical data. It explores the advantages of this approach over traditional wide-field radiotherapy and highlights the underlying mechanisms contributing to the observed pain relief. By addressing the limitations of existing treatments, this work may advance the management of bone malignancies and provide more effective and well-tolerated therapeutic options for patients (9-11).

Based on the high affinity of HEDP, a potent bisphosphonate, for bone tissue; the excellent properties of the MAX compound for chelating radionuclides; and its unique physicochemical characteristics, HEDP and MAX compounds were successfully synthesized using new synthetic methods (12-16) and then coupled with each other. MAX-HEDP was labeled with 99mTc under optimized labeling conditions. The choice of radiopharmaceutical is highly dependent on the patient's condition, such as renal function, bone marrow reserve, extent of cancer, and the physical properties of radionuclides (17). In addition, the commercial availability and affordability of radiopharmaceuticals must be considered (18).

The rhenium-188 (^188^Re) radionuclide has several advantages in this field. It can be extracted from 188W/^188^Re generators as sodium perrhenate, which is available on demand and is cost-effective (19). ^188^Re has a physical half-life of 16.9 hours and produces therapeutic beta rays with a maximum energy of 2.12 MeV, as well as imaging-friendly gamma rays with an energy of 155 keV. This dual capability enables visualization of the radioactive tracer distribution during treatment (20). Furthermore, ^188^Re shows higher bone uptake, more prolonged bone retention in vivo, and higher biological stability compared with ^188^Re-HEDP (^188^Re-2) over a 24-hour period.

## 2. Objectives

This study aimed to design, synthesize, radiolabel, and evaluate ^188^Re-MAX-HEDP as a novel bone-targeting theranostic radiopharmaceutical with potential diagnostic and therapeutic applications for bone-related diseases.

## 3. Methods

All chemicals used in this study were purchased from Merck, Germany, and used without further purification. For characterization of the prepared material, a Sartorius GCA 1603S/Max 320 g, d = 0.0001 g balance (Germany) and nuclear magnetic resonance (Bruker DRX-300 MHz; NMR) spectroscopy were used. In addition, inductively coupled plasma optical emission spectroscopy (ICP-OES) and an ultrasonic device (Elmasonic S 30H/SKU: 88900-C-2; power, 280 W; 220/240 V) were used to optimize the synthesis method. Furthermore, a MiniGita Star scanner (Raytest, Germany) was used for radio thin-layer chromatography (RTLC). Chromatographic analysis was performed using a Waters Alliance e2695 HPLC system (Waters, USA) equipped with a Raytest Gabi Star gamma detector (Raytest, Germany) and a Waters 2489 UV/Visible detector. Separation was achieved on a Waters XBridge C18 column (250 × 4.6 mm, 5 µm).

### 3.1. Synthesis of HEDP

HEDP was synthesized by condensation of acetic acid and phosphorus chloride. In a typical reaction, 90 g of acetic acid (1.50 mol) was mixed with 34.4 g of PCl_3_ (0.25 mol) and stirred at room temperature for 10 minutes. The mixture was cooled in an ice-water bath to control the exothermic reaction and gradually heated to 60 °C and then 110 - 120 °C over 20 - 30 minutes until phase separation occurred. Excess acetic acid was removed by steam distillation. The resulting semi-solid HEDP was cooled and stored (21, 22). The phosphate moiety of ^188^Re-HEDP is also adsorbed to calcium in bone hydroxyapatite. It is generally assumed that the ^188^Re-HEDP complex accumulates at sites of high osteoblastic activity. In other words, newly formed bone has a much larger surface area than established bone. In particular, the crystalline structure of hydroxyapatite in newly formed bone is amorphous and has a larger surface area than that in normal bone (23).

### 3.2. Synthesis of the MAX Chelator

**Figure 1.**
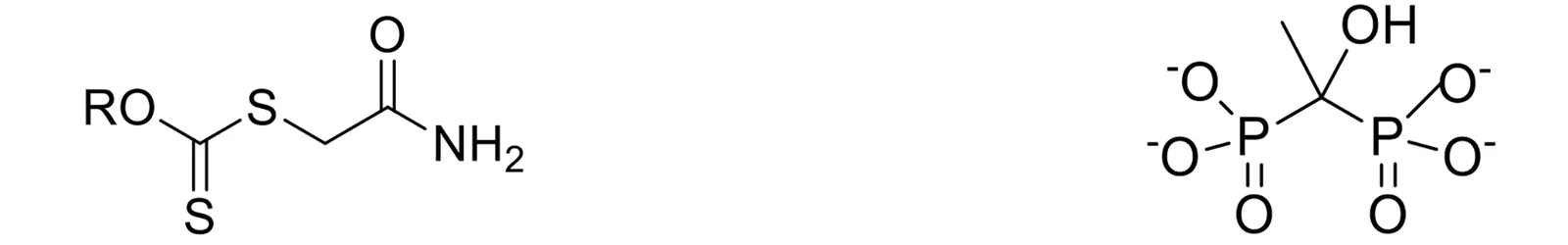
Schematic representation of the MAX chelator and HEDP structures, showing that MAX chelates rhenium, frees the phosphonate group on HEDP, and allows easier bone absorption.

MAX was synthesized from potassium ethyl xanthate, chloroacetamide, and DCM, followed by purification using sodium sulfate, petroleum ether, and ethyl acetate. The product was recrystallized and stored under a nitrogen atmosphere. Rhenium binds to the active phosphorus-containing sites on HEDP. This binding activity inhibits bone resorption. To improve HEDP bone uptake, the chelator MAX was added. MAX chelates rhenium, thereby freeing the phosphonate group on HEDP and enabling easier bone absorption. Figure 1 schematically shows the MAX chelator and HEDP structures.

### 3.3. Conjugation of MAX to HEDP

MAX was linked to HEDP using a DCC/NHS coupling strategy. The carboxylic group of MAX was activated with DCC to form an O-acylisourea intermediate, which was converted to an NHS ester by reaction with N-hydroxysuccinimide. HEDP, containing amine groups, acted as a nucleophile, forming a stable amide bond with the activated ester and yielding MAX-HEDP (24-26).

### 3.4. Radiolabeling of MAX-HEDP With ^188^ReO_4_Na^+^

To enhance labeling efficiency and prevent side reactions, fresh solutions of SnCl_2_ (1.5 mg in 1 mL of 0.1 M HCl) and ascorbic acid (0.75 mg in 1 mL water) were prepared immediately before the reaction and added to 5 mg of MAX-HEDP in 1 mL water. All reagents were accurately weighed using a digital scale, and solutions were measured with calibrated pipettes and graduated cylinders. The mixture was stirred using a magnetic stirrer. Subsequently, 10 mCi of ^188^Re (as ^188^ReO_4_Na^+^) in 150 µL was added, followed by 60 µL of acetate buffer to adjust the pH to approximately 7.0. The vial was heated at 95 °C for 30 minutes and filtered through a 0.22-µm sterile filter (27-30).

### 3.5. Stability Studies

The stability of the ^188^Re-MAX-HEDP complex was evaluated under multiple conditions to assess its integrity in various environments:

1) Physiological conditions: Incubation in phosphate-buffered saline (PBS; pH 7.4) at 25 °C and 37 °C.

2) Organic solvents: Incubation in methanol and acetone to evaluate stability in nonaqueous environments relevant to purification methods.

3) Biological matrix: Incubation in human serum at 37 °C to simulate in vivo conditions.

Aliquots were collected at predetermined time intervals. Radiochemical integrity was analyzed by instant thin-layer chromatography (ITLC) and high-performance liquid chromatography (HPLC), as detailed in Section 3.6. Stability was expressed as the percentage of the intact radio complex remaining over time (31).

### 3.6. Quality Control and Analytical Methods

Quality control of the ^188^Re-MAX-HEDP complex was performed using the following methods.

#### 3.6.1. Radiochemical Purity by Thin-Layer Chromatography

Radiochemical purity and labeling yield were determined by TLC. A 1-µL sample of the ^188^Re-MAX-HEDP complex was spotted on chromatography paper and developed using two mobile phases: methanol:acetone (1:1 v/v) and 0.9% saline. R_f_ values were determined precisely using a radio-TLC scanner (MiniGita Star, Raytest) with dedicated software that automatically detects peak centers and calculates migration distances.

#### 3.6.2. Radiochemical Purity by High-performance Liquid Chromatography

The radiochemical purity of the ^188^Re-MAX-HEDP complex was further analyzed by reverse-phase high-performance liquid chromatography (RP-HPLC). Analyses were performed using an Agilent 1260 series system equipped with a gamma-ray detector and a UV/Visible detector. Separation was achieved on a C18 column (250 × 4.6 mm, 5 µm) at ambient temperature. The mobile phase consisted of a gradient of 0.1% trifluoroacetic acid (TFA) in water (solvent A) and acetonitrile (solvent B) at a flow rate of 1.0 mL/minute. The injection volume was 20 µL.

#### 3.6.3. Other Quality Parameters

The pH of the final product was measured to ensure physiological compatibility. Endotoxin levels were determined using the Limulus amebocyte lysate (LAL) assay. Sterility was confirmed by incubation in thioglycolate and soybean-casein digest media for 14 days.

### 3.7. Biodistribution Studies

The in vivo biodistribution of ^188^Re-MAX-HEDP was evaluated in healthy adult male BALB/c mice (20 - 25 g). To assess the pharmacokinetic profile, as mentioned in Table 1, groups of mice (n = 5 per time point) received an intravenous injection of approximately 3.7 MBq (0.1 mCi) of the radiopharmaceutical in 100 µL of saline via the tail vein using a calibrated syringe. Animals were sacrificed at 1, 4, and 24 hours postinjection by euthanasia in a carbon dioxide chamber (Table 2). Following euthanasia, necropsy was performed. Key organs and tissues, including blood, heart, lungs, liver, spleen, kidneys, stomach, intestines, and bone (femur), were excised, cleaned of extraneous tissue, and weighed (32). Radioactivity in each sample was measured using a gamma counter. Tissue uptake was calculated and expressed as the percentage of the injected dose per gram of tissue (%ID/g). Data are presented as the mean %ID/g ± standard deviation (SD) for each group (n = 5).

**Table 1.** Administered and Absorbed Activity Parameters of ^188^Re-MAX-HEDP in the Mouse Biodistribution Experiment

Parameter	Value
**Activity in 5 mL volume of ^188^Re-MAX-HEDP**	1 mCi
**Activity injected in 100 µL of ^188^Re-MAX-HEDP**	0.25 mCi
**Absorbed activity in the mouse body**	80.57 µCi
**Weight of the tested mice**	30 g
**Weight of blood taken from mouse**	0.2 g

**Table 2.** Organ-Specific Radioactivity Counts and Absorption Percentages Following ^188^Re-MAX-HEDP Administration in Mice

Organ	Count	Absorption percentage
**Blood**	115	1.95
**Liver**	159	0.73
**Kidney**	136	0.45
**Stomach**	143	0.53
**Intestine**	173	0.91
**Bone**	181	1.01

## 4. Results

### 4.1. Synthesis and Characterization of the MAX-HEDP Ligand

The target ligand, MAX-HEDP (C_8_H_13_NNa_4_O_8_P_2_S_2_), was successfully synthesized and isolated as a white crystalline solid in an excellent yield of 91%. The compound exhibited a high melting point (> 300 °C), characteristic of ionic, thermally stable metal-chelating agents. The structure and purity of the ligand were unequivocally confirmed using a suite of spectroscopic and analytical techniques. The obtained product, HEDP-MAX, was identified by NMR spectroscopy. White crystals. Yield: 91%; MP ≥ 300 °C; 1H-NMR (D_2_O): 0.9 (s, 3H, CH_3_), 1.22 (t, 3H, CH_3_CH_2_), 3.58 (s, 3H, OCH_2_), 4.88 (s, H_2O), 3.99 (s, 2H, CH_2_S), 8.27 (s, 1H, NH); 31P-NMR (D_2_O): 19.76 (2 P; P-OH). IR (νmax, cm^-1^): 3500 (OH), 2800 - 2900 (NH), 2395 (P-H), 1650 (C = O), 1600 (O = P-O-H), 1250 (C-N), 1100 (CS_2_), 1140 (P = O).

#### 4.1.1. Nuclear Magnetic Resonance Spectroscopy

1H-NMR (D_2_O, 400 MHz): The spectrum confirmed the proposed molecular framework. Signals corresponding to aliphatic methyl (δ 0.9 and 1.22 ppm) and methoxy (δ 3.58 ppm) groups were present. A characteristic singlet for methylene protons adjacent to sulfur (δ 3.99 ppm, CH_2_S) and a broad singlet for an amide/protonated amine proton (δ 8.27 ppm, NH) were observed, confirming the incorporation of sulfur and nitrogen into the structure. The peak at δ 4.88 ppm was attributed to residual water (HOD) in the deuterated solvent.

31P-NMR (D_2_O, 108 MHz): A single, sharp resonance at δ 19.76 ppm was observed. This signal is definitive for phosphonate groups (-PO_3_^2-^) and indicates that the two phosphorus atoms in the molecule are chemically equivalent, consistent with a symmetric bisphosphonate core, a crucial feature for effective metal chelation.

#### 4.1.2. Fourier Transform Infrared Spectroscopy

The IR spectrum provided a functional group fingerprint. Key absorptions included a broad band at approximately 3500 cm^-1^ (O-H/N-H stretch), a P-H stretch at 2395 cm^-1^, a carbonyl (C = O) stretch at 1650 cm^-1^, and strong P = O stretching vibrations at 1140 cm^-1^. Bands corresponding to C-N (1250 cm^-1^) and C-S (1100 cm^-1^) stretches further validated the molecular scaffold containing nitrogen and sulfur atoms.

#### 4.1.3. High-Resolution Mass Spectrometry

Electrospray ionization mass spectrometry in negative mode provided definitive proof of the molecular formula and salt form. The spectrum was dominated by multiply charged ions, a hallmark of polyanionic compounds. The doubly charged ion [M-2Na+H]^2-^ observed at m/z 254.9581 and the triply charged ion [M-Na]^3-^ at m/z 163.6262 perfectly matched the theoretical isotopic distribution for C_8_H__13_NNa_4_O_8_P_2_S_2_. The observed mass accuracy and the pattern of sequential sodium loss confirm the compound's identity as a tetrasodium salt.

#### 4.1.4. Elemental Analysis

The elemental analysis results showed outstanding agreement with the theoretical composition calculated for C_8_H_13_NNa_4_O_8_P_2_S_2_, as summarized in Table 3. The minimal deviations for carbon (C), hydrogen (H), nitrogen (N), and sulfur (S) were within the instrumental margin of error, confirming both the proposed molecular formula and a high degree of chemical purity (> 98%) of the synthesized ligand.

**Table 3.** Elemental Analysis of MAX-HEDP

Element	Theoretical (%)	Found (%)	Difference (Δ%)
**Carbon (C)**	20.48	20.42 ± 0.15	-0.06
**Hydrogen (H)**	2.79	2.83 ± 0.10	+0.04
**Nitrogen (N)**	2.99	2.95 ± 0.12	-0.04
**Sulfur (S)**	13.67	13.60 ± 0.18	-0.07

The convergent data from 1H/31P NMR, FT-IR, HR-MS, and elemental analysis conclusively confirm the successful synthesis of the pure tetrasodium MAX-HEDP ligand with the correct molecular structure. The confirmed presence of the bisphosphonate motif, together with the nitrogen and sulfur donor atoms, validates its design as a suitable chelator for subsequent radiolabeling with rhenium-188 (^188^Re).

### 4.2. Quality Control of ^188^Re-MAX-HEDP

**Figure 2.**
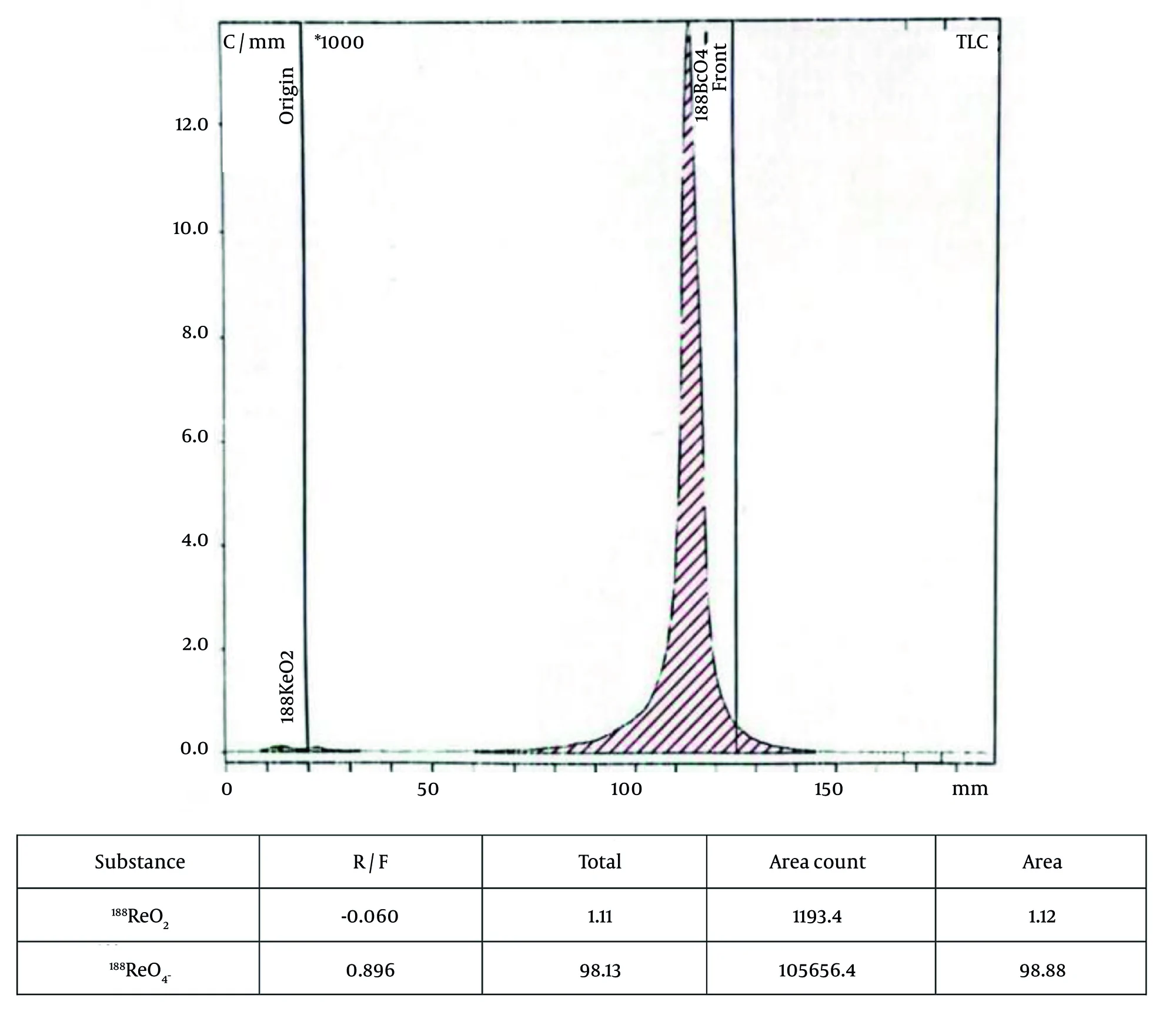
Chromatogram and integrated TLC data for free ^188^ReO_4_ in methanol:acetone as the mobile phase and Whatman paper as the stationary phase.

**Figure 3.**
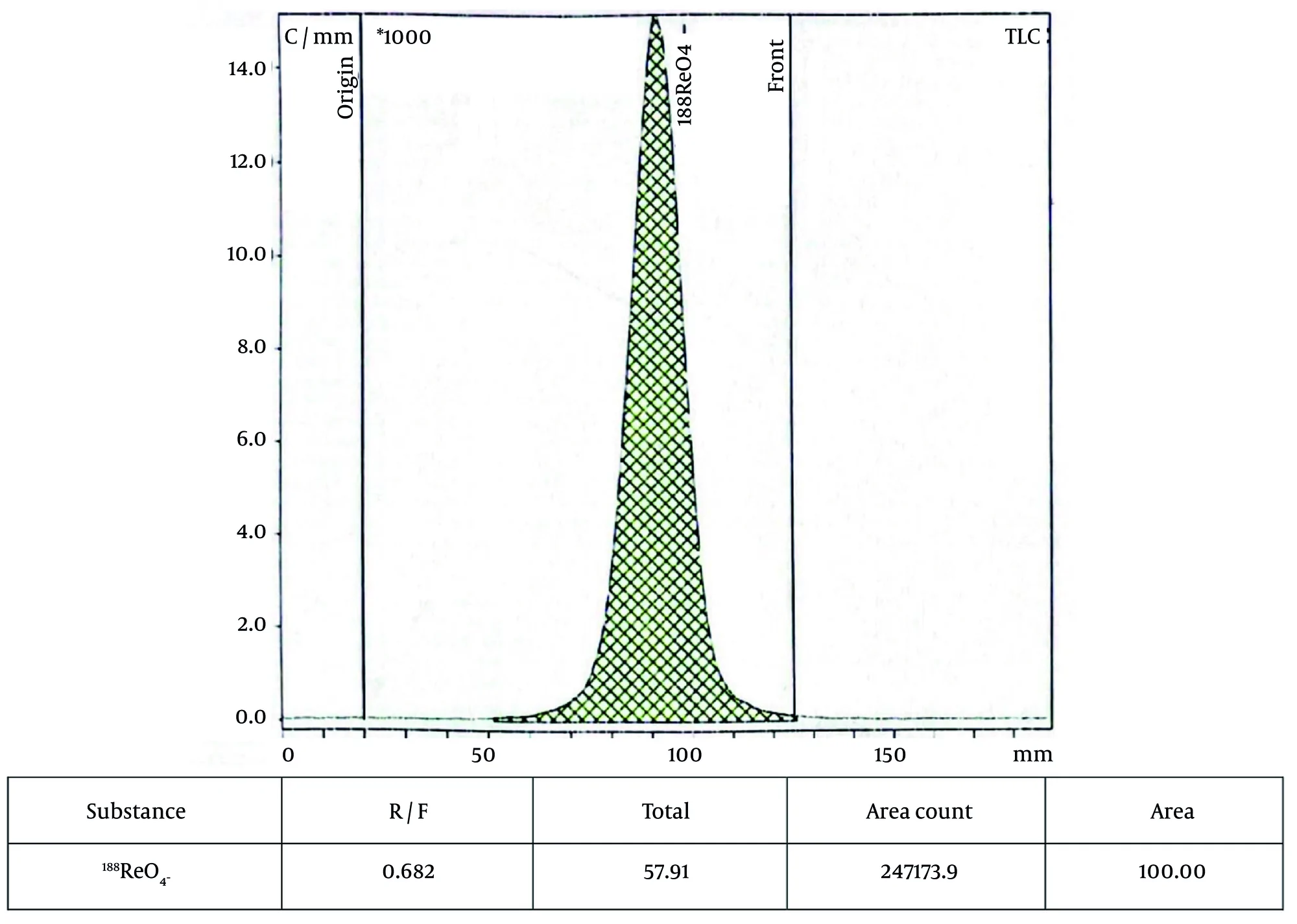
The plot and the data below illustrate ^188^ReO_4_^-^ chromatogram and Integration TLC of free 188Re in NaCl 0.9% as mobile phase and Whatman paper as stationary phase.

**Figure 4.**
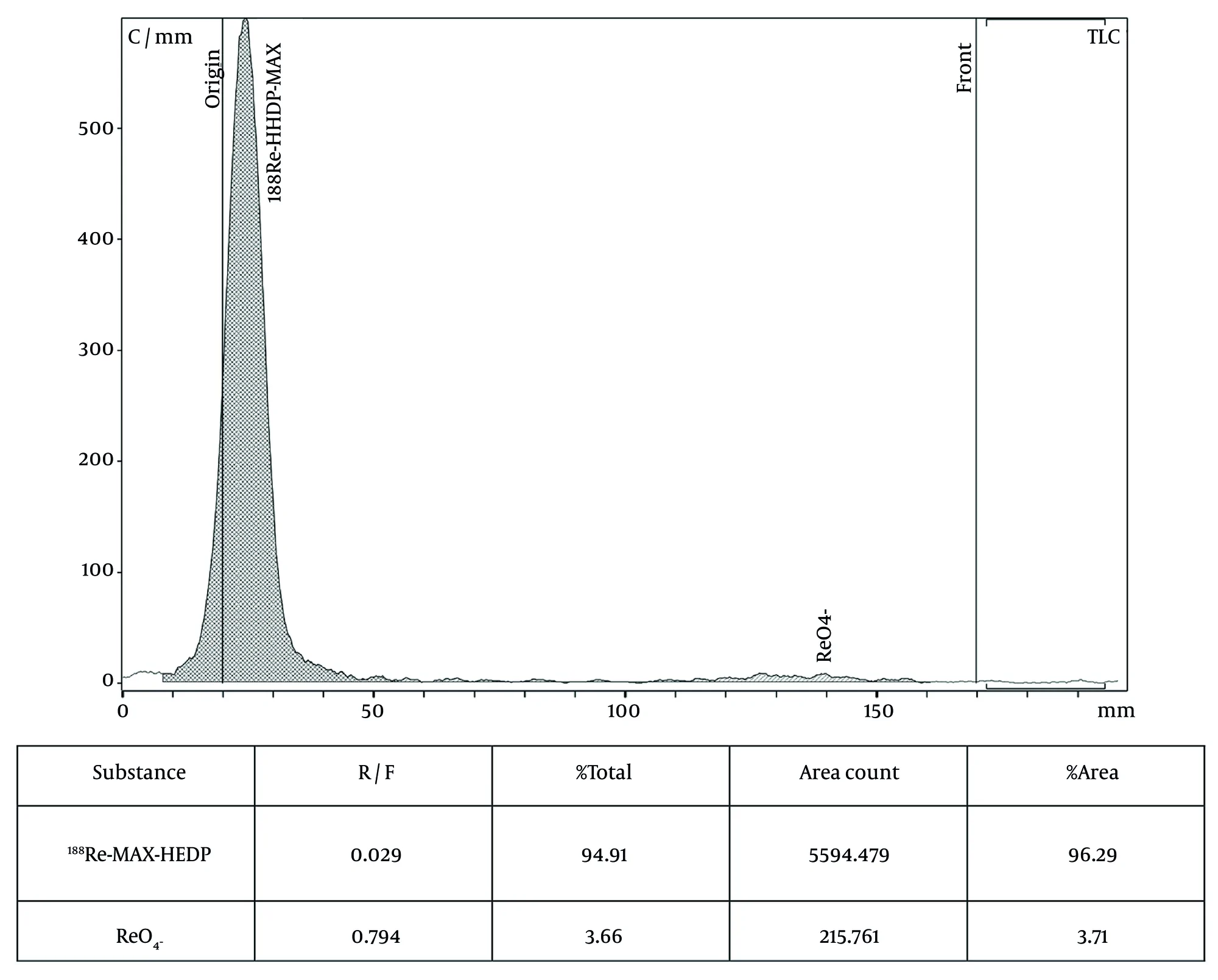
^188^Re-MAX-HEDP chromatogram in methanol:acetone solution as the mobile phase and Whatman paper as the stationary phase.

**Figure 5.**
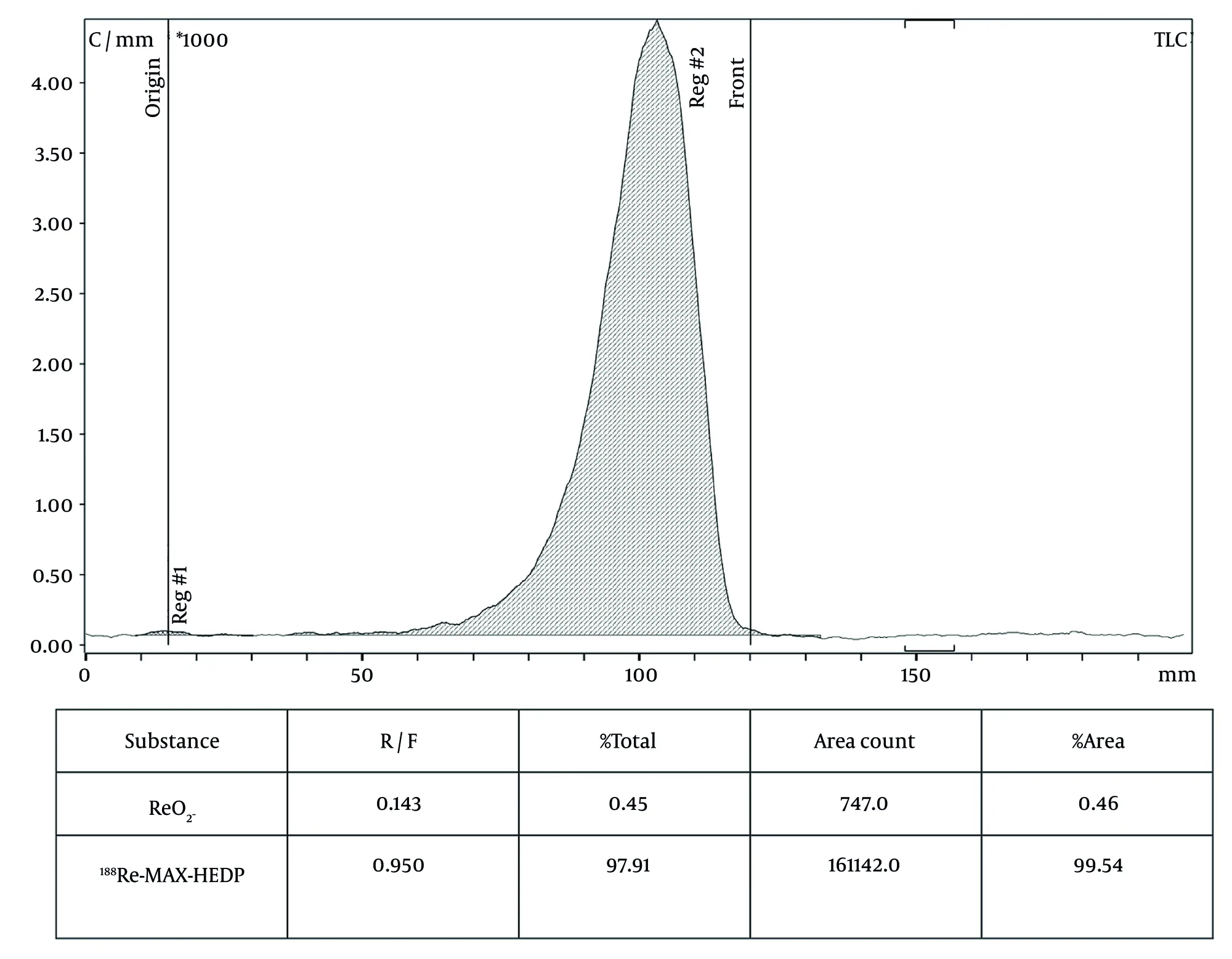
^188^Re-MAX-HEDP chromatogram in 0.9% saline solution as the mobile phase and Whatman paper as the stationary phase.

Preparing tin chloride (SnCl_2_) and ascorbic acid solutions immediately before labeling is essential to prevent side reactions and increase the yield, because these reagents can degrade under ambient conditions (27). The radiochemical purity of free ^188^ReO_4_^-^ in the final product was 0.28% and 0.21% using methanol:acetone and 0.9% saline as the mobile phase, respectively (Figures 2 and 3). The chromatogram in saline (Figure 3) showed only free ^188^ReO_4_^-^ without impurities. The radiochemical purity and labeling yield of the ^188^Re-MAX-HEDP complex were determined by chromatography (1-µL sample, paper, methanol:acetone/saline mobile phase), as shown in Figures 4 and 5. Finally, the stability of the labeled compound was assessed in human serum to simulate a biologically relevant environment.

#### 4.2.1. HPLC Method Specification for Radiochemical Purity of ^188^Re-MAX-HEDP

**Figure 6.**
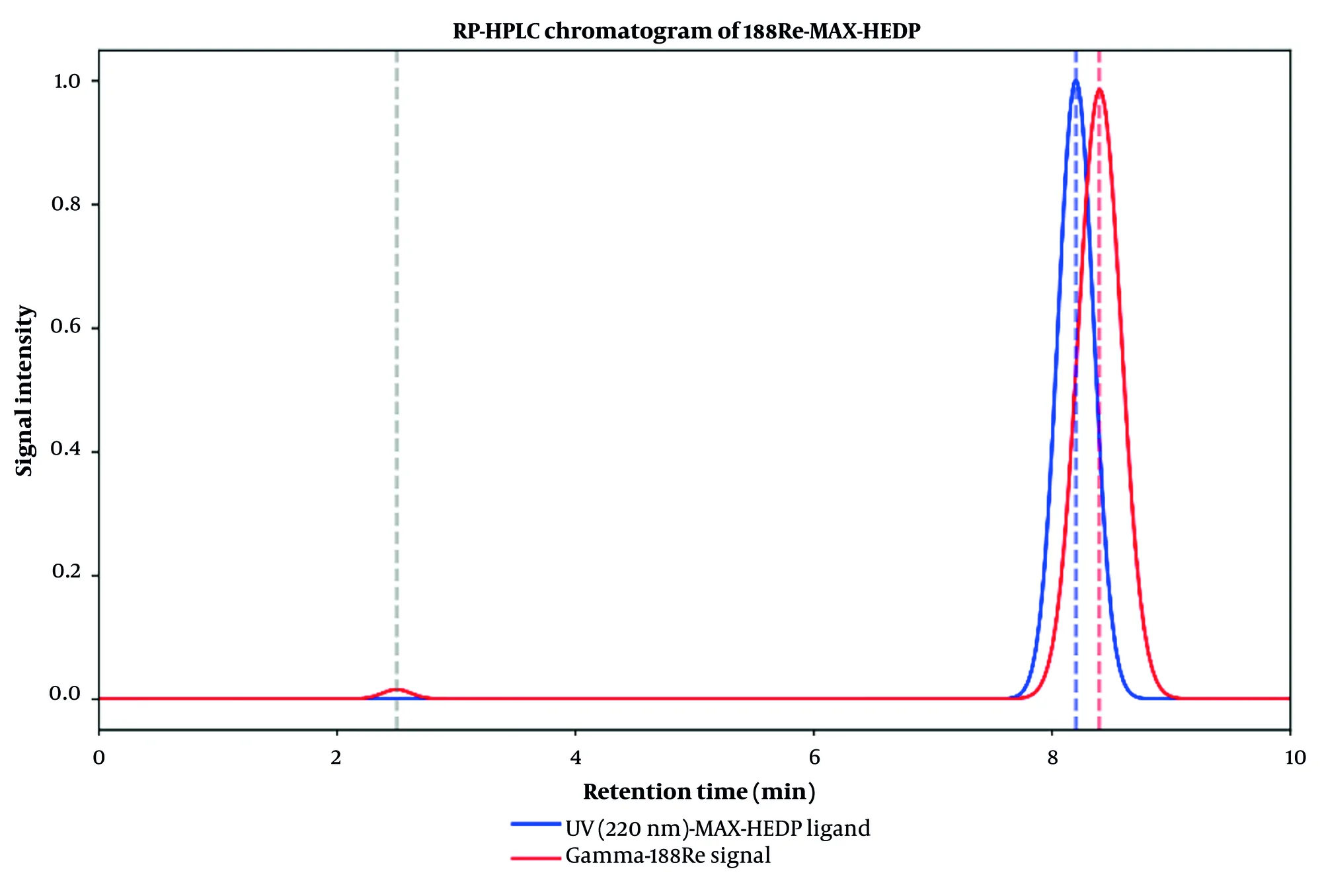
HPLC chromatogram of ^188^Re-MAX-HEDP.

The RP-HPLC chromatographic profile of ^188^Re-MAX-HEDP, as shown in Figure 6 and analyzed using dual detection (UV at 220 nm and gamma detection), confirms efficient radiolabeling and high radiochemical purity. The UV chromatogram shows a sharp MAX-HEDP ligand peak at R_t_ 8.1 - 8.2 minutes, whereas the gamma chromatogram exhibits a dominant co-eluting ^188^Re-MAX-HEDP peak at R_t_ 8.3 - 8.5 minutes (major peak at 8.4 minutes). A minor gamma peak at approximately 2.4 - 2.6 minutes corresponds to free perrhenate (^188^ReO_4_^-^), indicating only trace unbound radionuclide. The main radiocomplex peak accounts for 98.5 ± 0.5% radiochemical purity, calculated from gamma peak integration, with negligible impurity (< 5%). The close overlap between the UV and gamma signals confirms stable complex formation and intact ligand coordination after radiolabeling. System suitability criteria were met, demonstrating good peak shape, baseline stability, and clear separation between the complex and free perrhenate. Overall, these results confirm the successful synthesis of ^188^Re-MAX-HEDP with high purity and suitability for further biological and preclinical evaluation as a bone-targeting radiopharmaceutical.

### 4.3. Labeling Efficiency and Stability Study

The labeling efficiency of ^188^Re to MAX-HEDP was measured by ascending RTLC using Whatman sheets (Whatman, NJ, USA). RTLC was performed using a 1-µL sample of the final fraction, spotted onto a chromatography paper strip and developed in methanol:acetone and 0.9% saline solutions as the mobile phases. Labeling efficiency was calculated using the following equation (28):

Labelingefficiency%=Totalcounts-countsoffree188ReTotalcounts×100

In the RTLC chromatogram, the horizontal axis (x) represents the advancement rate of each species in millimeters, whereas the vertical axis indicates the count value in millimeters. These measurements were used to derive additional parameters. For instance, the R_f_ value was determined using the horizontal axis. During the counting process, both the spotting point and the solvent advance point (in millimeters) were identified. Based on these points, the retention factor for each species was calculated and reported as the primary parameter in RTLC. The vertical axis enabled computation of the area under the peak for each separated species by integrating the count value per millimeter. This area was then used to assess the radiochemical purity of the sample. The spectra obtained using each mobile phase are shown in Figures 2-6.

### 4.4. Stability Testing of Radiolabeled ^188^Re-MAX-HEDP in Human Serum

**Figure 7.**
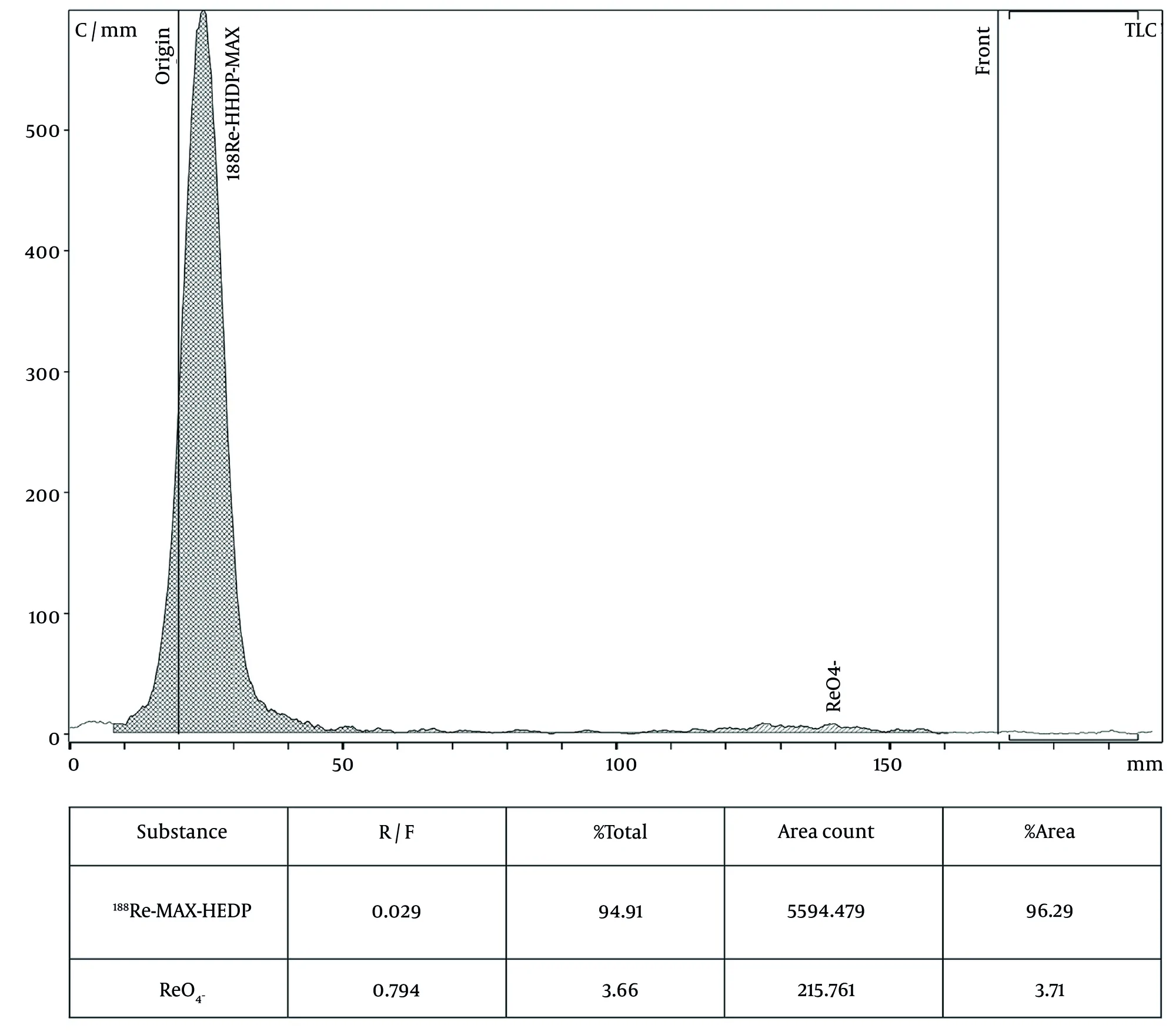
Stability chromatogram of ^188^Re-MAX-HEDP after 20 hours. Evaluation of biological stability revealed that the compound remained highly stable, retaining more than 96% integrity under ambient conditions and undergoing less than 10% degradation at 37 °C.

For the serum stability assessment, 10 mL of human blood was centrifuged at 6000 rpm (4032 relative centrifugal force [RCF], or g-force) for 20 minutes, and 1 mL of serum was collected. Then, 100 µL of ^188^Re-MAX-HEDP was added to 1 mL of serum and incubated at 37 °C for up to 20 hours. Labeling efficiency and radiochemical purity were assessed. Figure 7 and the data below illustrate the stability testing under biological conditions and showed excellent results: at room temperature, the compound retained more than 96% integrity, and even at 37 °C, it showed minimal degradation, with less than a 10% reduction.

### 4.5. Biodistribution

**Figure 8.**
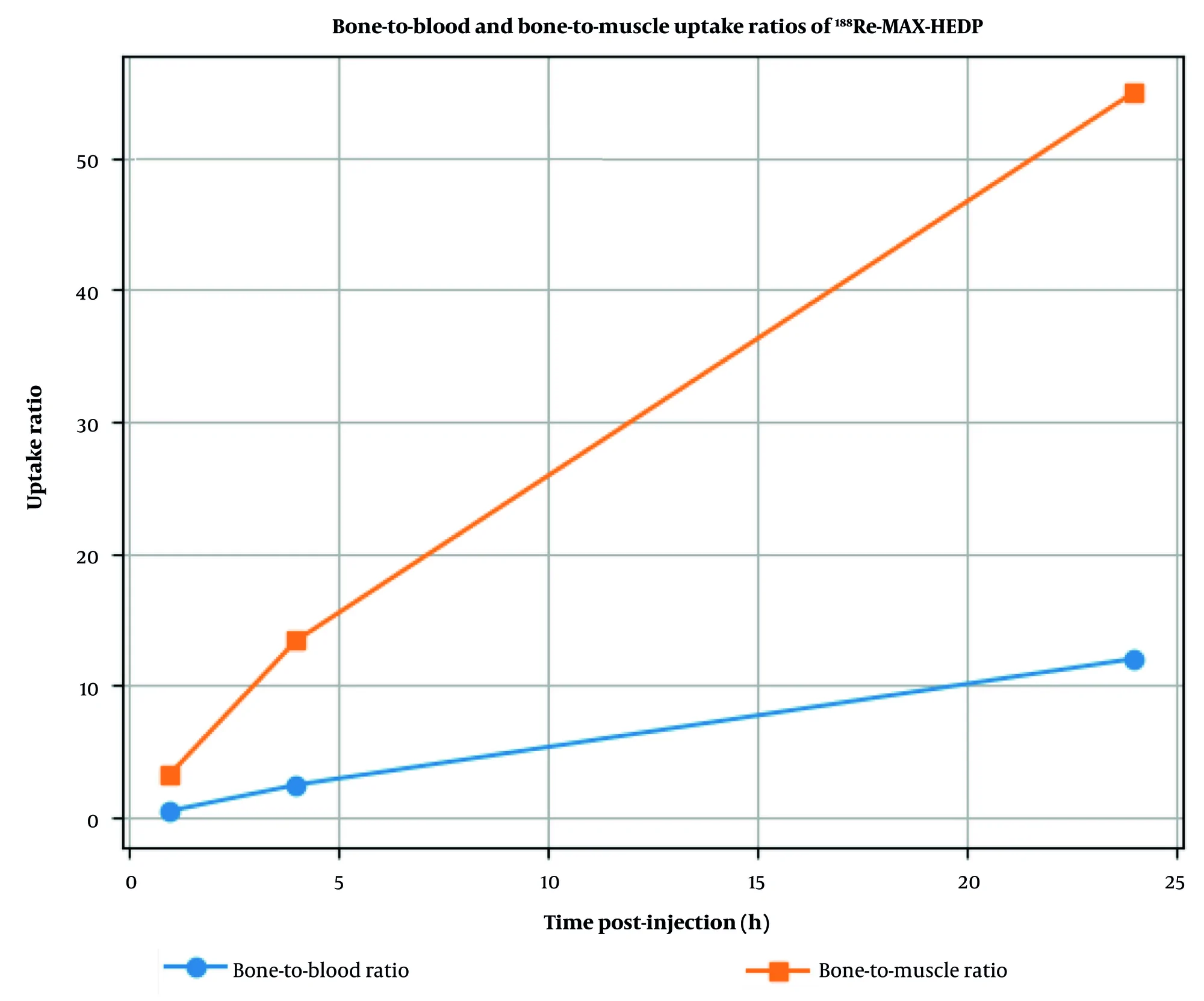
Bone-to-blood and bone-to-muscle uptake ratios of ^188^Re-MAX-HEDP. The complex exhibits a time-dependent pattern of skeletal accumulation, with early blood-phase activity observed at 1 hour postinjection (p.i.), followed by swift clearance from the vascular compartment and soft tissues.

The in vivo biodistribution profile of ^188^Re-MAX-HEDP confirms its effective bone-seeking behavior and favorable pharmacokinetics, supporting its potential as a therapeutic agent for skeletal metastases. As shown in Figure 8, the complex exhibits a characteristic time-dependent pattern of skeletal accumulation. Although initial blood activity is observable at 1 hour postinjection (p.i.), reflecting the circulation phase, rapid clearance from blood and soft tissues occurs. Concurrently, significant and progressive uptake in bone is observed, reaching 2.41 ± 0.36%ID/g at 4 hours and 3.85 ± 0.52%ID/g at 24 hours. This pharmacokinetic profile results in excellent target-to-background ratios, a critical metric for therapeutic efficacy. Both the bone-to-blood and bone-to-muscle uptake ratios increase substantially over time (Figure 7). The rising bone-to-blood ratio indicates efficient blood clearance and selective retention in bone. Similarly, the increasing bone-to-muscle ratio demonstrates high specificity for osseous tissue over general soft tissue, which is essential for minimizing off-target radiation exposure.

The biodistribution kinetics of ^188^Re-MAX-HEDP align with the established profile of bisphosphonate-based radiopharmaceuticals, such as ^188^Re-HEDP, in which maximal bone uptake is typically achieved several hours after administration. This confirms that integration of the MAX chelator successfully enhances the complex's stability in vivo without impairing the innate affinity of the HEDP moiety for hydroxyapatite, the mineral component of bone.

## 5. Discussion

The biodistribution data demonstrate that ^188^Re-MAX-HEDP possesses the defining characteristics of an effective bone-targeting therapeutic agent: specific and sustained accumulation in skeletal tissue, rapid clearance from nontarget organs, and consequently, high target-to-background ratios. These properties underscore its suitability for the targeted radiotherapy of osteoblastic lesions and bone metastases.

### 5.1. Conclusions

The present study reports the successful synthesis, radiolabeling, and preclinical evaluation of ^188^Re-MAX-HEDP as a novel bone-seeking theranostic radiopharmaceutical. The MAX-HEDP ligand was synthesized with a high chemical yield and radiolabeled efficiently with ^188^Re, achieving radiochemical purities exceeding 98%. Stability studies in saline, organic solvents, and human serum confirmed excellent in vitro robustness of the radiocomplex under physiological conditions. In vivo biodistribution studies in BALB/c mice demonstrated time-dependent skeletal accumulation, with a progressive increase in bone uptake and concomitant clearance from blood and soft tissues. The resulting high bone-to-blood and bone-to-muscle ratios at later time points are consistent with the pharmacokinetic behavior of clinically established bisphosphonate-based radiopharmaceuticals, such as ^188^Re-HEDP. These findings confirm that incorporation of the MAX chelator enhances complex stability while preserving the hydroxyapatite affinity of the HEDP moiety. Overall, the biological performance of ^188^Re-MAX-HEDP supports its potential application as a bone-targeted therapeutic radiopharmaceutical for the management of skeletal metastases and other bone-related malignancies. Further investigations, including dosimetry, toxicity, and therapeutic efficacy studies in disease models, are warranted to advance this agent toward clinical translation.

## Data Availability

The dataset presented in the study is available on request from the corresponding author during submission or after publication.
